# Supplementary material for: Revealing the Phenotypic and Genomic Background for PHA Production from Rapeseed-Biodiesel Crude Glycerol Using Photobacterium ganghwense C2.2
Source: Int J Mol Sci. 2022 Nov 9;23(22):13754. doi: 10.3390/ijms232213754 (PMC9697146; doi:10.3390/ijms232213754)
Supplement: Supplementary file 1 [file ijms-23-13754-s001.zip › ijms-1966453-supplementary.pdf]

Supplementary material for

Revealing phenotypic and genomic background for PHA production from rapeseed-biodiesel crude glycerol using *Photobacterium ganghwense* C2.2

Irina Lascu[1,**], Ana Maria Tănase[1,*,**], Piotr Jablonski[2], Iulia Chiciudean[1], Maria Preda[1], Sorin Marius Avramescu[3], Knut Irgum[2], Ileana Stoica[1]

[1] Department of Genetics, Faculty of Biology, University of Bucharest, Bucharest, Romania.

[2] Department of Chemistry, Faculty of Science and Technology, Umeå University, Umeå, Sweden

[3] Department of Organic Chemistry, Biochemistry and Catalysis, Faculty of Chemistry, University of Bucharest, Bucharest, Romania.

* Correspondence: ana-maria.tanase@bio.unibuc.ro.

** These authors contributed equally to this work.

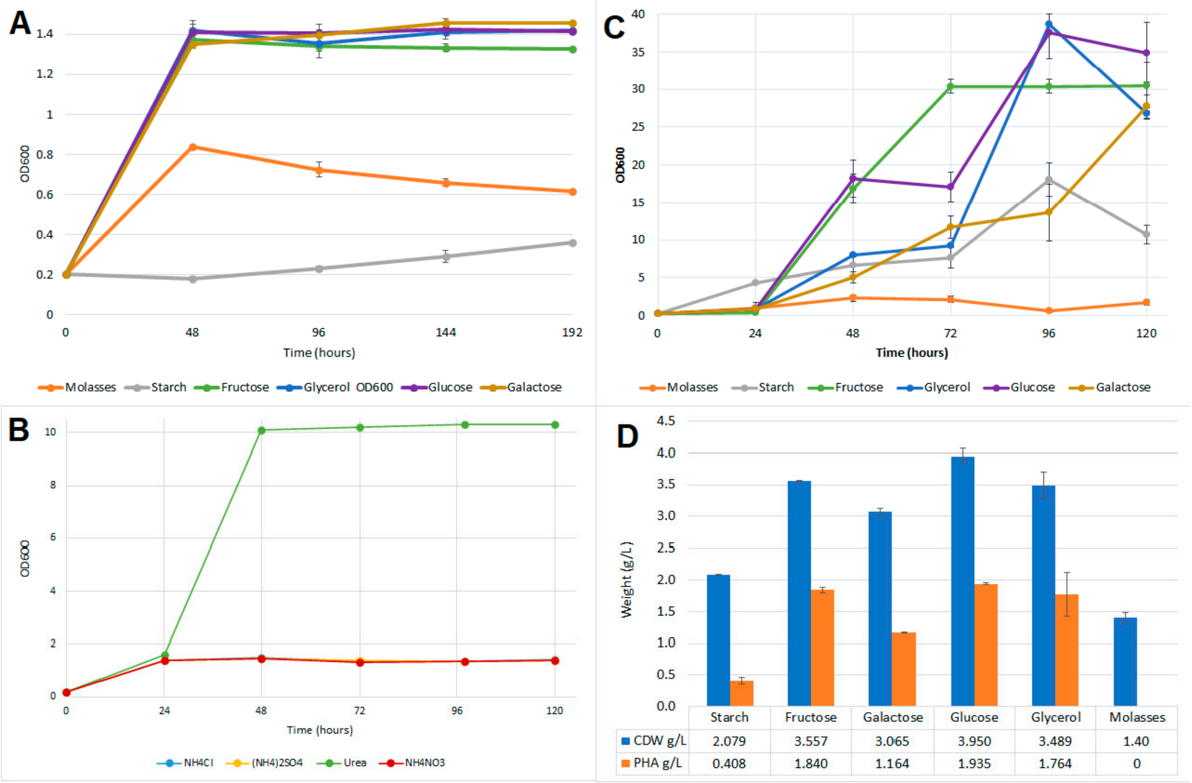

Figure S1. Growth and PHA production of strain C2.2 using various carbon and nitrogen sources. (A) Evolution of optical density values throughout cultivation using ammonium chloride as a nitrogen source and various carbon sources. (B) Strain growth using fructose as a carbon source and various nitrogen sources. (C) Testing of various carbon sources using urea as a nitrogen source. (D) Average CDW and PHA content for strain C2.2 when cultivated using urea and various carbon sources, after 120 h.

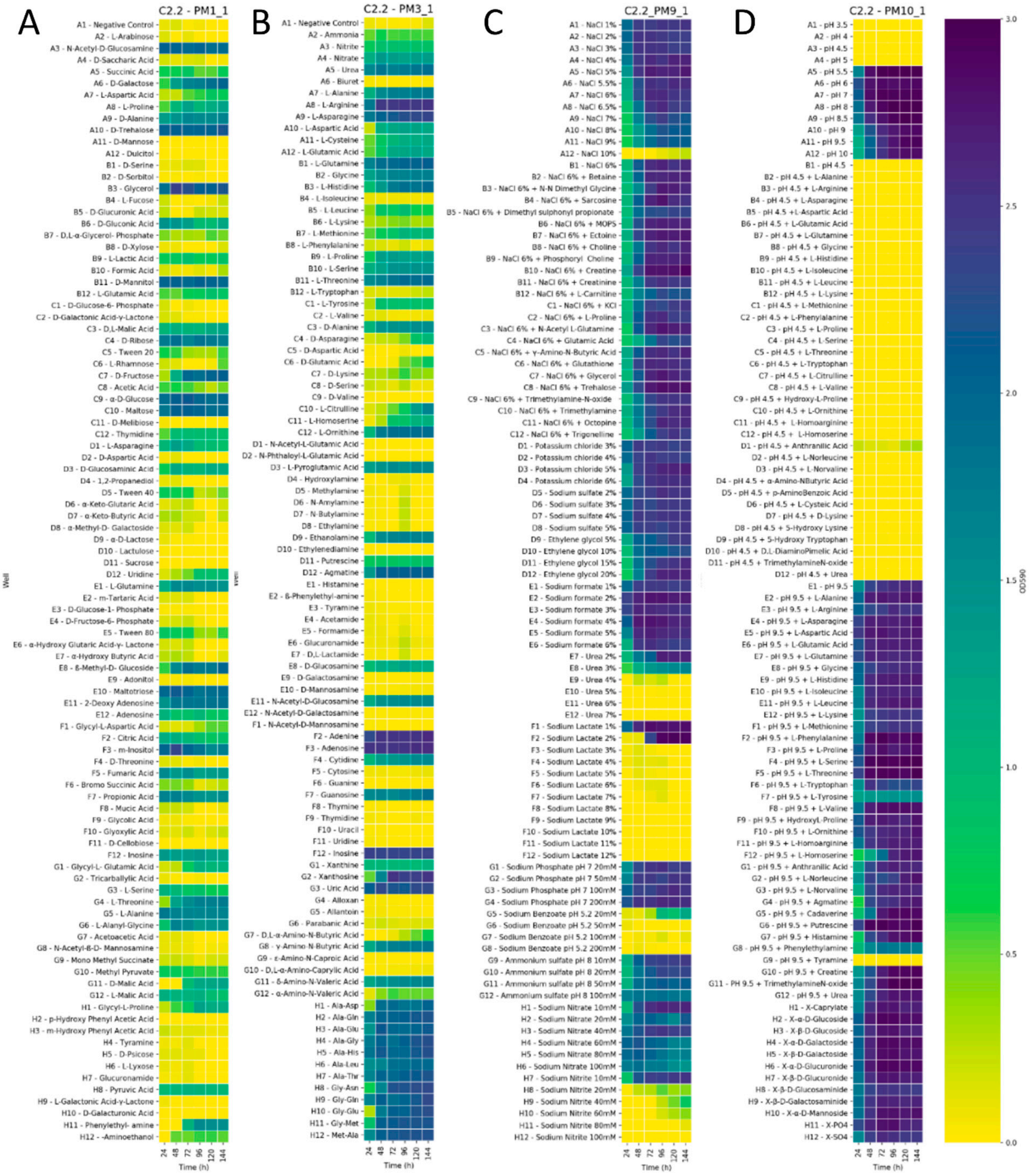

Figure S2. Heatmap representation of strain C2.2 OD_{590} values in Biolog tests using plates PM1 (A), PM3 (B), PM9 (C), and PM10 (D), throughout the six days of cultivation. Wells are listed in the order A1-H12.

Table S1. Genes involved in various metabolic pathways annotated in strain C2.2 complete genome.

Metabolic pathway	Gene/enzyme/protein name	Translated gene ID
**Urea ABC transporter**	*urtA* – urea ABC transporter substrate-binding protein	WP_047884448.1
	urtB – urea ABC transporter permease subunit UrtB	WP_047884827.1
	urtC – urea ABC transporter permease subunit UrtC	WP_107193837.1
	urtD – urea ABC transporter ATP-binding subunit UrtD	WP_207104232.1
	urtE – urea ABC transporter ATP-binding subunit UrtE	WP_047884447.1
**Urease**	*ureD* – urease accesory protein UreD	WP_084712504.1
	ureA – urease subunit gamma	WP_047884446.1
	ureB – urease subunit beta	WP_207104081.1
	ureC – urease subunit alpha	WP_207104082.1
	ureE – urease accessory protein UreE	WP_207104083.1
**Glycine betaine synthesis**	Choline dehydrogenase	WP_047885691.1
	Aldehyde dehydrogenase	WP_047885109.1
		WP_047885485.1
**Na^+/K^+ - H^+ antiporters**	*nhaP2*	WP_207104222.1
	nhaA	WP_047886478.1
		WP_207103231.1
	nhaB	WP_047884003.1
	nhaC	WP_047884030.1
	nhaD	WP_084712508.1
**Fatty acid degradation**	*atoB*	WP_047884439.1
		WP_047885796
	fadD	WP_047883241.1
		WP_047883493.1
		WP_047883703.1
		WP_047885973.1
		WP_207103586.1
	fadE	WP_047884083.1
		WP_047886809.1
	2,3-dehydroadipyl-CoA hydratase	WP_084712533.1
	fadB	WP_207103416.1
	fadJ	WP_047887366.1
	fadA	WP_047887367.1
	fadI	WP_047887523.1
**Methanol tolerance**	*katG* – catalase-peroxidase	WP_047883481.1

**Formaldehyde oxidation**	II	S-(hydroxymethyl)glutathione dehydrogenase	WP_207103312.1
		S-formylglutathione hydrolase	WP_207103313.1
	VII	*folD* methenyltetrahydrofolate cyclohydrolase	WP_011219298.1
		formyltetrahydrofolate deformylase	WP_207103524.1
**Fatty acid biosynthesis, type II**		*accC*	WP_047885270.1
		fabD	WP_047883398.1
		fabH	WP_047883397.1
			WP_047885535.1
		fabY	WP_047884431.1
		fabB	WP_047883770.1
			WP_207103912.1
		fabF	WP_047883401.1
		fabG	WP_047883399.1
			WP_047884888.1
			WP_047885051.1
			WP_047885790.1
		fabA	WP_047886715.1
		fabZ	WP_047883171.1
		fabV	WP_047884084.1
			WP_047885041.1
**Short/medium chain acyl-CoA synthesis**		*acs*	WP_047886168.1
			WP_207103377.1
		prpE	WP_047884612.1
		acsA	WP_107193900.1
**Fatty acid desaturation**		stearoyl-CoA desaturase	WP_047885932.1
**Coenzyme A separation from fatty acids**		*tesA*	WP_047887426.1
		tesB	WP_047887354.1
		yciA	WP_047883312.1
